# Research on Shape Memory Properties of PETG Based on 4D-Printed Negative Poisson’s Ratio Structures

**DOI:** 10.3390/polym18091039

**Published:** 2026-04-24

**Authors:** Zepeng Liu, Shaogang Liu, Bai Chen

**Affiliations:** 1 College of Mechanical Engineering, Tianjin University of Science and Technology, Tianjin 300457, China; liuzepeng1999@mail.tust.edu.cn (Z.L.); 25802930@mail.tust.edu.cn (B.C.); 2 Key Laboratory of Integrated Design and On-Line Monitoring for Light Industry & Food Machinery and Equipment, College of Mechanical Engineering, Tianjin University of Science and Technology, Tianjin 300457, China

**Keywords:** 4D printing, negative Poisson’s ratio structure, PETG, shape memory performance, response surface methodology

## Abstract

This research systematically investigates the shape memory properties of re-entrant hexagonal negative Poisson’s ratio (NPR) honeycomb structures fabricated via 4D printing, using polyethylene terephthalate glycol (PETG) and polylactic acid (PLA) as comparative materials. Periodic honeycomb models with varied wall thicknesses and structural unit angles were designed, and their effects on shape recovery time and recovery rate were examined. Response surface methodology (RSM) based on a Box–Behnken design was employed to optimize key process parameters, including the wall thickness, structural unit angle, and mold pressing angle. The results demonstrate that PETG exhibits significantly superior shape memory performance compared to PLA, characterized by a shorter recovery time and higher recovery rate under thermal stimulation. Through RSM optimization, the optimal parameter combination was identified as a wall thickness of 0.5 mm, a structural unit angle of 65°, and a mold pressing angle of 135°, which was subsequently validated experimentally, demonstrating a high degree of consistency between predicted and actual outcomes. This study not only clarifies the influence of the structural parameters on the shape memory behavior of NPR honeycomb systems but also provides parameter guidance and a practical experimental basis for the application of PETG in 4D-printed intelligent structures, with potential implications for soft robotics, aerospace, and biomedical devices.

## 1. Introduction

The aim of 4D printing technology is to develop new smart materials capable of actively changing their original form in response to external stimuli [1] such as temperature, magnetic field, humidity, and light. Unlike traditional 3D-printed objects, which have static, immutable structures [2] and cannot adapt to dynamic demands, 4D printing is an advanced manufacturing that introduces the time dimension. According to the ISO/ASTM 52900 standard [3], 4D printing is defined as using 4D printing technology to fabricate objects that can undergo shape, property, or functional changes under preset stimuli [4]. Through intelligent material and structure design, 4D printing grants the printed object controllable shape or function evolution in response to external stimuli; i.e., the object possesses sufficient dynamic adaptability to realize self-construction following a change in the external environment [5,6,7,8]. It is this kind of active structure regulation ability that has driven researchers to further explore how to combine 4D printing with macroscopic structures with special mechanical behaviors to expand their performance boundary into different application scenarios. Negative Poisson’s ratio structures, due to their counter-intuitive deformation characteristics and excellent mechanical properties, are ideal for achieving structure–function integration via 4D printing technology [9,10].

Poisson’s ratio (ν) is the negative ratio of transverse strain to axial strain. Most natural materials have a positive Poisson’s ratio (0~0.5), meaning they contract laterally when stretched. The core feature of auxetic materials is a negative Poisson’s ratio. The auxetic effect specifically refers to the phenomenon wherein a structure expands laterally when subjected to uniaxial tension and contracts laterally when compressed. This counter-intuitive deformation behavior stems from its carefully designed microscopic topology, such as re-entrant, chiral, rotating polygons, etc., rather than the material itself [11,12,13,14]. Compared to other traditional structural designs, negative Poisson’s ratio structures demonstrate significant advantages due to their unique tensile expansion characteristics, such as stronger indentation resistance, superior impact resistance, more efficient energy absorption, better vibration damping, and lightweight characteristics [15,16]. In the meantime, traditional manufacturing methods face difficulties in processing these complex microstructures; however, 4D printing technology employs layer-by-layer material deposition to accurately fabricate metamaterials with specific negative Poisson’s ratio effects. It also allows for the flexible adjustment of unit parameters, enabling the active design and programming of mechanical properties such as Poisson’s ratio and stiffness. The organic integration of 4D printing technology with negative Poisson’s ratio structures achieves performance that surpasses traditional materials [17,18]. It also provides revolutionary solutions for fields such as aerospace [19], biomedical engineering [20], flexible electronics [21], and advanced protective equipment [22]. This combination represents an important direction for the development of a structurally integrated design and smart materials [23].

Based on the above advantages, in recent years, research on the application of negative Poisson’s ratio structures in various fields has rapidly expanded and yielded fruitful results.

The integration of negative Poisson’s ratio structures with 4D printing has enabled a wide range of applications across various engineering fields. In the aerospace sector, Nian et al. [24] designed a nature-inspired 3D-printed multi-gradient aerospace NPR meta-structure, which demonstrated excellent lightweight performance and high energy absorption capacity, showing broad application prospects in spacecraft buffering and vehicle protection. Similarly, Wagih et al. [25] successfully designed and validated a 3D-printed metal stiffener with NPR flanges, significantly improving the strength, toughness, and lightweight level of stainless steel–carbon fiber reinforced polymer T-joints. In biomedical engineering, Hu et al. [26] integrated NPR porous structures into 3D-printed custom hemipelvic prostheses, demonstrating good tumor control, functional recovery, and radiographic integration effects in reconstruction after resection of pelvic malignant tumors. For flexible electronics and sensors, Choi et al. [27] fabricated a piezoresistive sensor based on 3D-printed thermoplastic polyurethane (TPU) coated with castor oil-based waterborne polyurethane/graphene NPR structure. The sensor exhibited high sensitivity, good repeatability, and stable resistance change detection during compression. In protective equipment and energy absorption applications, Shen Luyu et al. [28] studied the buffer performance of NPR honeycomb structures based on 3D-printed TPU material, using multi-objective optimization to significantly enhance energy absorption performance for ballistic helmet liners. Alomarah et al. [29] constructed two types of three-dimensional auxetic metamaterials through geometric biomimicry based on butterfly contours, effectively balancing the contradiction between the auxetic effect and energy absorption. These studies collectively demonstrate the tremendous potential of combining negative Poisson’s ratio structures with 4D printing technology. However, research gaps remain regarding the negative Poisson’s ratio structural properties of specific high-performance printing materials under 4D printing, especially for new bio-based polyester materials such as polyethylene terephthalate glycol (PETG).

PETG is one such material that has gained increasing interest in additive manufacturing due to its excellent transparency, biocompatibility, processability, and hydrolysis resistance [30,31,32]. As a bio-based polyester, PETG also aligns with growing environmental sustainability requirements. However, existing studies on PETG have predominantly concentrated on filament modification, composite reinforcement, and printing accuracy optimization [33,34,35], leaving a significant gap in understanding its 4D printing potential, especially in comparison with established materials like PLA for NPR-based smart structures. While Bouguermouh et al. have recently begun exploring 4D-printed auxetic structures using nanocellulose-reinforced PLA/PETG blends [36], systematic investigations of pure PETG’s shape memory performance in NPR configurations remain scarce, and the influence of structural parameters on its thermomechanical response is not yet well-established. This study aims to address this gap by systematically investigating the shape memory properties of re-entrant hexagonal NPR honeycomb structures fabricated via 4D printing using PETG as the primary material, with PLA serving as a benchmark for comparison. The specific objectives are (1) to design and fabricate periodic NPR honeycomb structures with varying wall thicknesses and structural unit angles; (2) to evaluate the effects of key geometric parameters—including wall thickness, structural unit angle, and mold pressing angle—on shape recovery time and recovery rate; (3) to compare the shape memory performance of PETG and PLA under identical test conditions; (4) to optimize process parameters using response surface methodology (RSM) based on a Box–Behnken design; and (5) to validate the optimal parameter combination through experimental testing. The findings of this work provide a practical experimental basis for the application of PETG in 4D-printed intelligent structures and offer parametric guidance for the design of NPR-based smart components in fields such as soft robotics, aerospace engineering, and biomedical devices.

## 2. Design and Analysis of Re-Entrant Hexagonal Honeycomb Structure Workpiece

**Figure 1:**
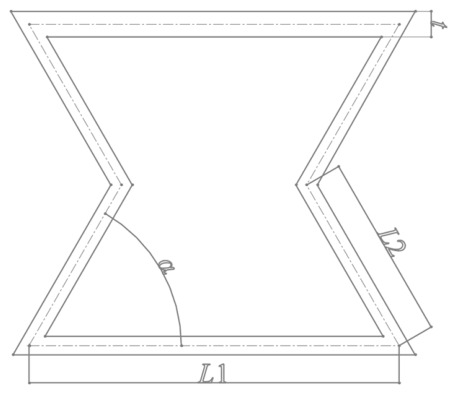
Schematic diagram of cellular geometric parameters.

A re-entrant hexagonal honeycomb structure with an overall shape resembling an hourglass is designed. Its cellular geometric parameters are shown in Figure 1. Here, *L*_1_ is the length of the horizontal cellular wall, fixed at 10 mm; *L*_2_ is the length of the inclined cellular wall, fixed at 5 mm; the angle *α* between the horizontal and inclined cellular walls is the structural unit angle; and *t* is the wall thickness, which is the same for both horizontal and inclined cellular walls.

### 2.1. Research on Negative Poisson’s Ratio

A re-entrant shape is not a sufficient condition for a structure to have a negative Poisson’s ratio; this only occurs when the structural unit angle satisfies certain conditions. Therefore, to generate a negative Poisson’s effect, Equation (1) must be satisfied.

(1) $$\frac{L_{1}}{L_{2}}\geq 2\;\cos\alpha$$

The larger the negative Poisson’s ratio, the more pronounced the auxetic effect. Therefore, the negative Poisson’s ratio is an important parameter affecting the mechanical performance of the workpiece structure. The specific calculation formula for the negative Poisson’s ratio of the re-entrant hexagonal honeycomb structure is given by Equation (2).

(2) $$\upsilon =\frac{\sin\alpha \;\tan\alpha }{\frac{L_{1}}{L_{2}}+\cos\alpha }\frac{1-{\left(\frac{t}{L_{2}}\right)}^{2}}{1+\tan^{2}\alpha {\left(\frac{t}{L_{2}}\right)}^{2}}$$

**Figure 2:**
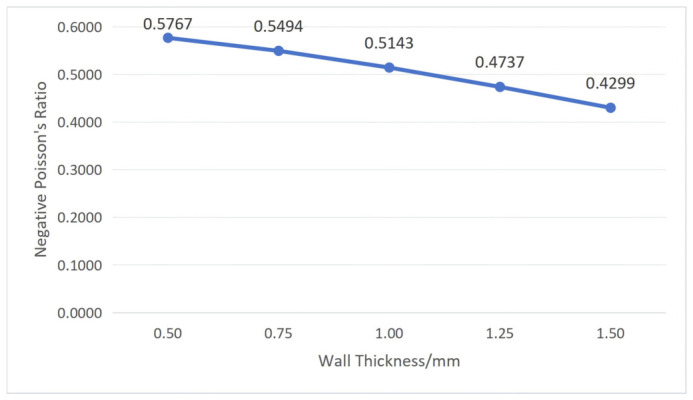
The influence of wall thickness on negative Poisson’s ratio (structural unit angle = 60°).

**Figure 3:**
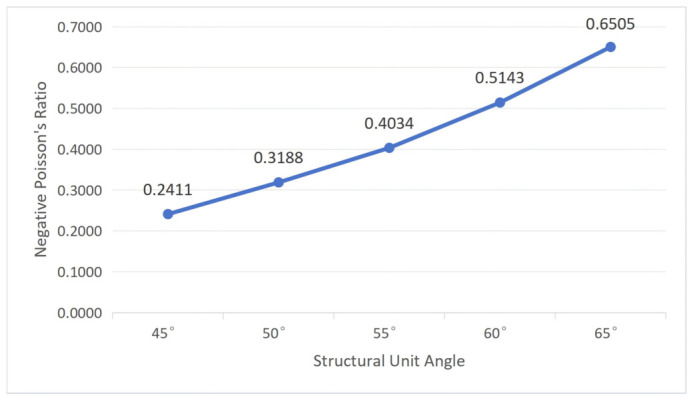
The influence of structural unit angle on negative Poisson’s ratio (wall thickness = 1.00 mm).

According to Equation (2), when *L*_1_ and *L*_2_ are constant, the negative Poisson’s ratio depends only on *α* and *t*. To understand the influence of cellular geometric parameters on the negative Poisson’s ratio, first, fix the structural unit angle *α* = 60°; then, substitute wall thickness *t* values of 0.50 mm, 0.75 mm, 1.00 mm, 1.25 mm, and 1.50 mm, respectively. Then, fix wall thickness *t* = 1.00 mm and substitute *α* values of 45°, 50°, 55°, 60°, and 65°, respectively. The calculation results are shown in Figure 2 and Figure 3.

From Figure 2 and Figure 3, it can be concluded that the negative Poisson’s ratio of the re-entrant hexagonal honeycomb structure decreases with increasing wall thickness and increases with increasing structural unit angle. However, the influence of wall thickness on the negative Poisson’s ratio is not evident, with small variation amplitude, which is essentially negligible. Therefore, the negative Poisson’s ratio of the re-entrant hexagonal honeycomb structure is mainly influenced by the size of the structural unit angle.

### 2.2. Research on Relative Density

Besides negative Poisson’s ratio, for periodic multi-cellular structures, relative density is also an important parameter for evaluating whether their structural mechanical performance is excellent. For two-dimensional periodic structures, relative density is expressed as the ratio of the area occupied by all wall thicknesses in a cellular to the characteristic area of the cellular, as shown in Equation (3).

(3) $$\rho_{c}=\frac{S_{a}}{S_{0}}$$

In Equation (3), *ρ_c_* is the relative density of negative Poisson’s ratio hexagonal honeycomb structure, *S_a_* is the area occupied by all wall thicknesses in the cellular, and *S*_0_ is the characteristic area of the cellular. Specifically for the re-entrant hexagonal honeycomb structure selected in this experiment, it can be expressed as follows—Equation (4):

(4) $$\rho_{c}=\frac{t}{2L_{2}}\times \frac{\frac{L_{1}}{L_{2}}+2}{\cos\;\alpha \;\left(\frac{L_{1}}{L_{2}}+\sin\;\alpha \right)}$$

**Figure 4:**
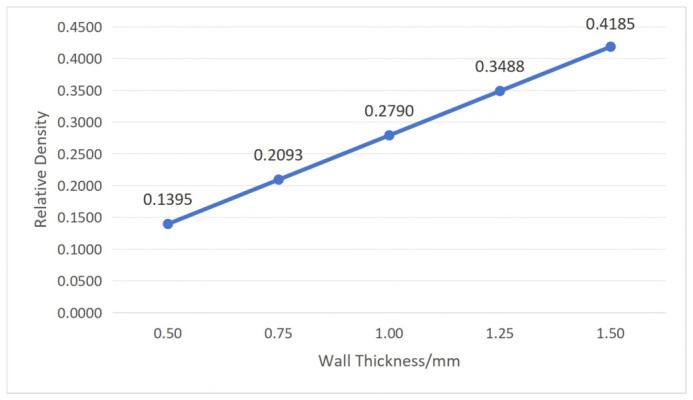
The influence of wall thickness on relative density (structural unit angle = 60°).

**Figure 5:**
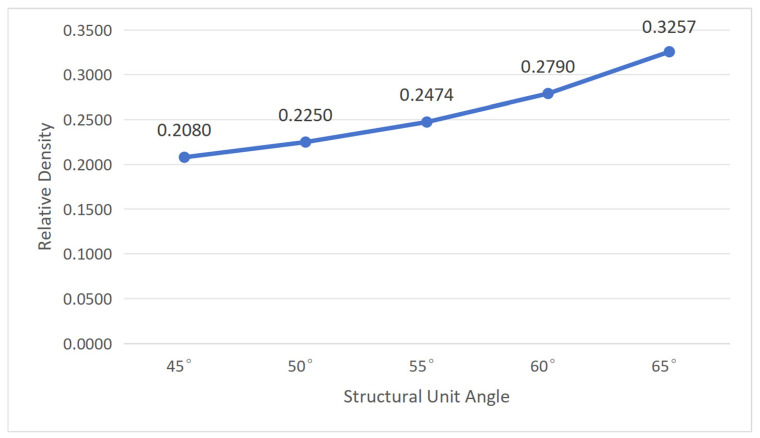
The influence of structural unit angle on relative density (wall thickness = 1.00 mm).

Through Equations (3) and (4), it can be seen that when *L*_1_ and *L*_2_ are constant, the relative density depends only on *α* and *t*. To understand the influence of cellular geometric parameters on relative density, first, fix the structural unit angle *α* = 60°; then, substitute wall thickness *t* values of 0.50 mm, 0.75 mm, 1.00 mm, 1.25 mm, and 1.50 mm, respectively. Then, fix wall thickness *t* = 1.00 mm and substitute α values of 45°, 50°, 55°, 60°, and 65°, respectively. The calculation results are shown in Figure 4 and Figure 5.

From Figure 4 and Figure 5, it can be concluded that the relative density of the re-entrant hexagonal honeycomb structure increases with increasing wall thickness and increases with increasing structural unit angle. However, the influence of the structural unit angle on relative density is not evident, with small variation amplitude, which is essentially negligible. Therefore, the relative density of the re-entrant hexagonal honeycomb structure is mainly influenced by the wall thickness.

## 3. Experimental Section

### 3.1. Raw Materials

PETG filament: PETG Basic, red, diameter 1.75 ± 0.03 mm, density 1.25 g/cm^3^, China Shenzhen Bambu Lab Technology Co., Ltd (Shenzhen, China).

PLA filament: PLA Basic, green, diameter 1.75 ± 0.03 mm, density 1.24 g/cm^3^, China Shenzhen Bambu Lab Technology Co., Ltd.

### 3.2. Instruments and Equipment

3D Printer: Bambu Lab P1S, BambuLab, China Shenzhen Bambu Lab Technology Co., Ltd.

Stopwatch: Shanghai Diamond Brand Mechanical Stopwatch Diamond JM-803, China Shanghai Watch Factory (Shanghai, China).

Protractor: Deli Stainless Steel Angle Ruler Combination Universal High-Precision Protractor, China Deli Group Co., Ltd. (Ningbo, China).

Differential Scanning Calorimeter: Mettler Differential Scanning Calorimeter, China Mettler-Toledo (Shanghai, China).

Scanning Electron Microscope: Thermo Fisher Field Emission Electron Microscope FEI450, China Thermo Fisher Scientific Co., Ltd. (Shanghai, China).

### 3.3. Printing Parameters

The parameters of the FDM (Fused Deposition Modeling) 3D printer were adjusted according to the properties of PETG and PLA materials, as detailed in Table 1.

### 3.4. Fabrication of Negative Poisson’s Ratio Honeycomb Structure Workpiece

**Figure 6:**
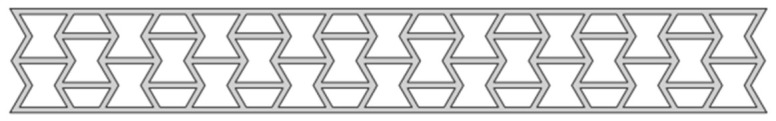
Two-dimensional schematic diagram of printed negative Poisson’s ratio honeycomb structure.

Using SolidWorks 2020 software, following the principle of periodic arrangement with respect to the wall thickness and structural unit angle, the cellulars were arranged according to a certain pattern. Then, the height was set to 10.00 mm, and 3D modeling was completed using the extrude boss feature. The model was then saved in the STL format, imported into Bambu Studio software V2.4.0.70 for slicing, and the final product printing was completed (Figure 6).

**Figure 7:**
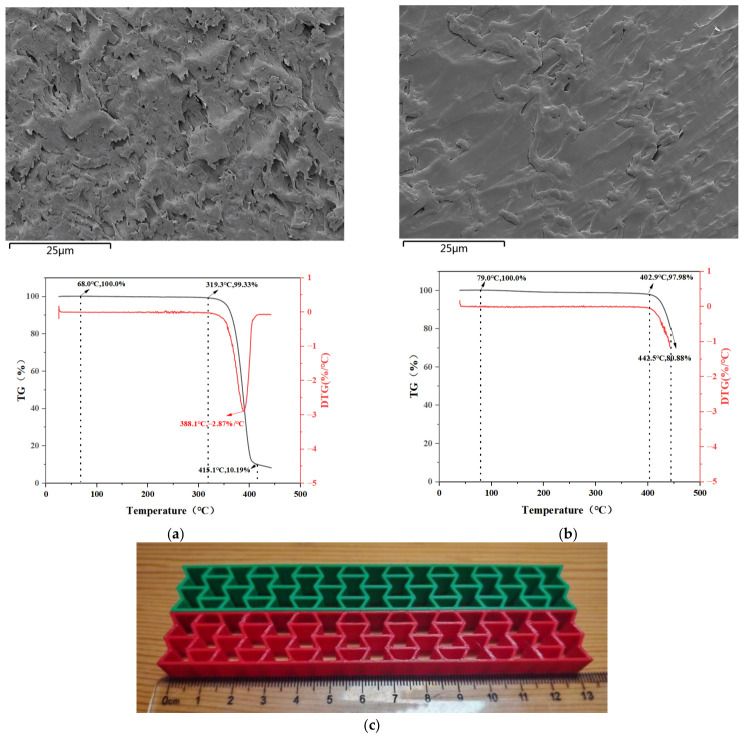
Basic information of workpiece immersed in PLA and PETG. (**a**) Electron microscope scanning diagram of PLA and differential scanning calorimetry of glass transition temperature. (**b**) Electron microscope scanning diagram of PETG and differential scanning calorimetry of glass transition temperature. (**c**) Physical image of negative Poisson’s ratio honeycomb structure print (wall thickness = 1 mm; structural unit angle = 60°).

As shown in Figure 7, scanning electron microscopy (SEM) and differential scanning calorimetry (DSC) were performed on the printed specimens made of PLA and PETG, respectively. The glass transition temperature (*T_g_*) of PLA was determined to be 68 °C, which is consistent with the manufacturer’s calibrated value of 67 ± 5 °C. The *T_g_* of PETG was determined to be 79 °C, which is consistent with the manufacturer’s calibrated value of 80 ± 5 °C.

### 3.5. Experimental Principle and Process

Shape memory polymers (SMPs) are a class of smart materials with programmable temporary shapes and fixed permanent shapes. Their 4D printing behavior is based on the thermodynamics of glass transition or melting transition [37]. SMPs are made from various materials, including thermoplastic polymers, elastomers, and composites. A typical thermoplastic SMP undergoes the following three-stage process: programming, fixing, and recovery. Above *T_g_*, the material is in a high elastic state and can be deformed into a temporary shape by applying external force; after cooling below *T_g_*, the molecular chain segments freeze, and the temporary shape is fixed; upon reheating above *T_g_*, the molecular chain segments thaw, and entropy elasticity drives the material to recover its initial shape [38].

**Figure 8:**
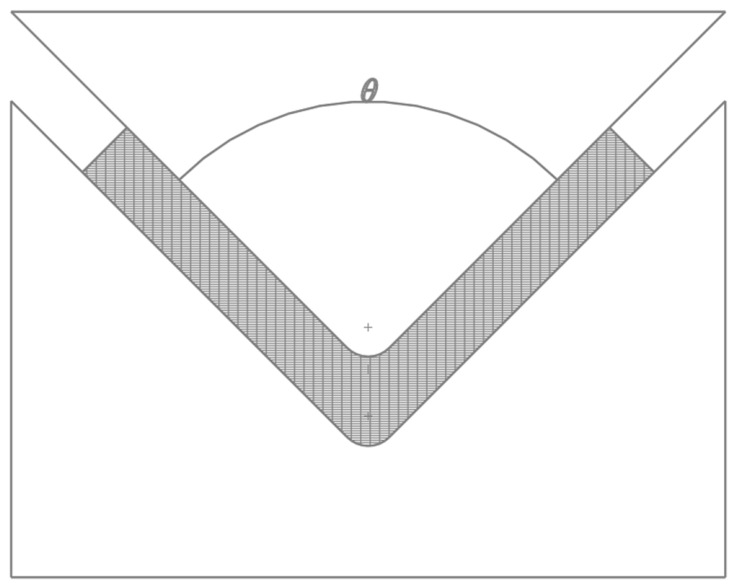
Schematic diagram of compression mold (The shaded part represents the workpiece).

Based on the response mechanism of thermoplastic SMPs, the specific experimental process consists of the following steps: first, use the 3D printer to fabricate the workpiece. Second, completely immerse the workpiece in a water bath of PLA at 70 °C and of PETG at 85 °C (which is above the *T_g_* of both materials) for 90 s. Then, quickly remove the workpiece, place it in a mold for bending and shaping, as shown in Figure 8, fix it at room temperature for at least 60 s to obtain the temporary shape, and place it in 20 °C cooling water for sufficient cooling to solidify this temporary shape. Finally, immerse the bent workpiece again completely in a water bath at the same temperature as the heating temperature. The thermally driven effect will gradually restore it to its initial morphology, and the recovery time is recorded upon immersion.

In this study, FDM was used solely to fabricate the permanent shape of negative Poisson’s ratio honeycomb structure. The 4D printing functionality was achieved through a post-printing thermomechanical programming process, which involves heating the printed workpiece above its glass transition temperature, deforming it into a temporary shape using a mold, cooling to fix the temporary shape, and finally reheating it to trigger shape recovery. This two-stage approach (additive manufacturing of permanent shape followed by thermomechanical programming) constitutes the complete 4D printing cycle.

In the pre-experiment, it is found that the shape recovery mainly occurs in the early and middle stage of immersion in the water bath, and the final angle tends to be stable. Therefore, the recovery rate calculated by the final residual angle can effectively characterize the overall recovery capacity of the workpiece. After the experiment, use a high-precision protractor to measure the residual angle *β* of the recovered workpiece, conduct multiple experiments to obtain the average value, and calculate the shape recovery rate *R* of the workpiece accordingly. The specific calculation method is shown in Equation (5).

(5) $$R=\frac{180{}^{\circ}-\beta }{180{}^{\circ}}\times 100\%$$

## 4. Results Analysis and Discussion

### 4.1. Influence of Bending Deformation Point on Workpiece Shape Memory Performance

**Figure 9:**
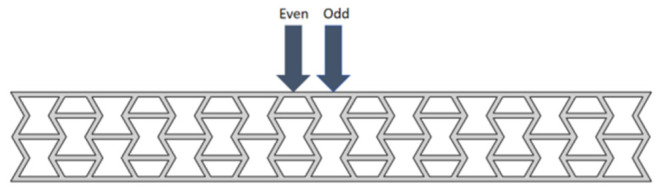
Position of bending deformation point.

For structures with multi-period regular arrangement, because the position of the bending deformation point changes its geometric morphology and stress distribution, thereby affecting the overall functional performance, the bending deformation point during mold pressing is one of the key factors affecting the shape memory performance of this structure. At this point, the position of the bending deformation point is crucial. Because the workpiece prepared in this experiment is obtained by repeating a single cellular 9 times, through the point of applied pressure and the parity of the structural units, the bending deformation point located at the very center of the workpiece, i.e., the position of the fifth cell repetition, is named the odd point. The bending deformation point adjacent to the odd point, i.e., the position of the fourth or sixth cellular repetition, is named the even point, as shown in Figure 9.

**Figure 10:**
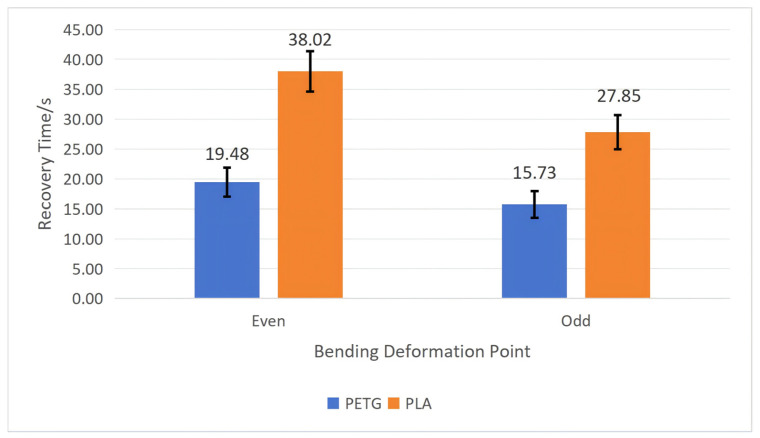
The influence of bending deformation points on the recovery time of the workpiece (structural unit angle = 60°; wall thickness = 1 mm; and mold pressing angle = 90°). Error bars represent the standard deviation (*n* = 5).

**Figure 11:**
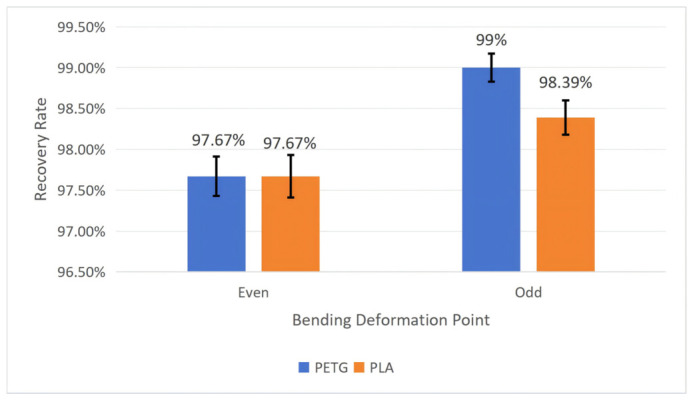
The influence of bending deformation points on the recovery rate of the workpiece (structural unit angle = 60°; wall thickness = 1 mm; and mold pressing angle = 90°). Error bars represent the standard deviation (*n* = 5).

To test the influence of the bending deformation point on the workpiece’s shape memory performance, we fixed the wall thickness *t* at 1 mm, the structural unit angle *α* at 60°, and the mold pressing angle *θ* at 90°. Tests were conducted on PETG and PLA workpieces, respectively, recording their recovery time and calculating their shape recovery rate according to Equation (5). After repeating each experiment five times, we calculated the average and standard deviation. The specific results are shown in Figure 10 and Figure 11.

As can be seen from Figure 10 and Figure 11, the performance results of workpieces made from both PETG and PLA materials are the same; i.e., the average recovery rate and average recovery time are better when the bending deformation point is an odd point than an even point. Additionally, the average recovery rate for the PETG material at the odd point is greater than that for the PLA material, and regardless whether the points are odd or even, the average recovery time for the PETG material is shorter than that for the PLA material.

The fundamental reason for this phenomenon is that for negative Poisson’s ratio honeycomb structures, their mechanical performance greatly depends on the symmetry of the load [39]. When the load acts on the geometric center, the symmetry of the structure is maintained, and the deformation force is transmitted evenly through the internal network to all cellulars, allowing the elastic strain energy stored in each cellular to be similar. In contrast, an eccentric load induces bending moments, causing the strain on the compression side to be much larger than on the opposite side. This strain gradient causes some cellulars (especially those near the load point) to enter plastic yield prematurely or undergo local fracture, reducing the stored elastic strain energy and increasing irreversible deformation. Moreover, under water bath heating, heat is conducted from the outside to the inside. When the center is compressed, uniform thermal expansion and modulus decrease help to release residual stress synchronously, promoting overall cooperative recovery. After eccentric compression, residual stress is uneven, and during the heating process, the recovery driving forces in different regions vary, with regions restraining each other, ultimately leading to incomplete overall recovery.

To prevent load asymmetry from affecting the experimental results, the workpieces used in subsequent tests all had their bending deformation point set at the odd point at their geometric center.

### 4.2. Influence of Wall Thickness on Workpiece Shape Memory Performance

The size of the cellular wall thickness is a core factor affecting the lightweight effect of the overall structure. Optimizing wall thickness can significantly improve the strength, stiffness, and stability of the structure, thereby achieving an optimal balance between the mechanical performance of the workpiece and material consumption.

**Figure 12:**
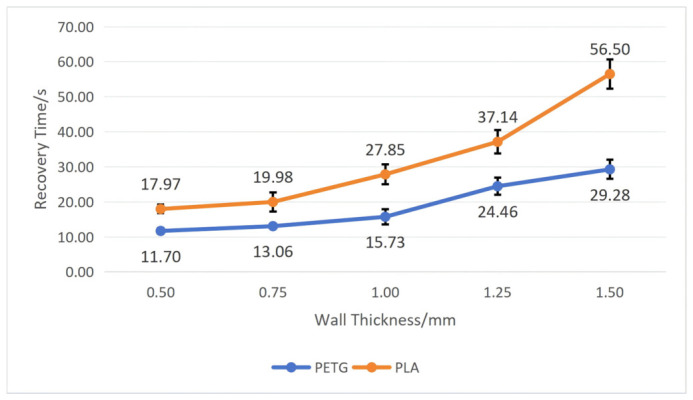
The influence of wall thickness on the recovery time of the workpiece (structural unit angle = 60°; mold pressing angle = 90°). Error bars represent the standard deviation (*n* = 5).

**Figure 13:**
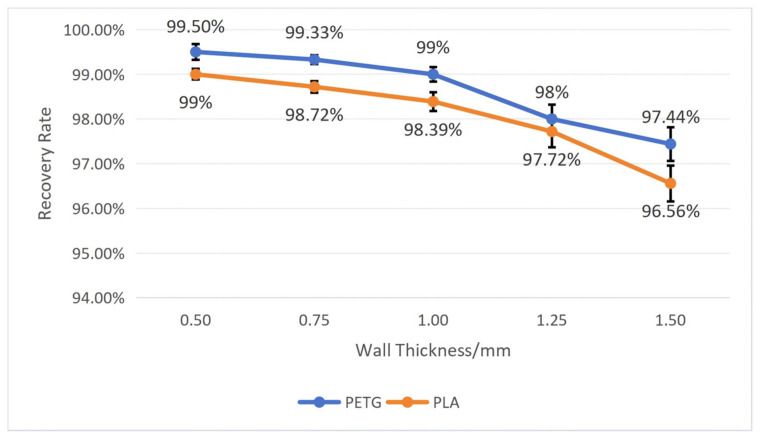
The influence of wall thickness on the recovery rate of the workpiece (structural unit angle = 60°; mold pressing angle = 90°). Error bars represent the standard deviation (*n* = 5).

To test the influence of wall thickness on the workpiece’s shape memory performance, we fixed the structural unit angle *α* at 60 degrees and the mold pressing angle *θ* at 90°. By comparing workpieces with five different wall thicknesses, namely, 0.50 mm, 0.75 mm, 1.00 mm, 1.25 mm, and 1.5 mm, tests were conducted on PETG and PLA workpieces, respectively, recording their recovery time and calculating their shape recovery rate according to Equation (5). After repeating each experiment five times, we calculated the average and standard deviation. The specific results are shown in Figure 12 and Figure 13.

As shown in Figure 12 and Figure 13, both materials exhibit roughly the same trend: as wall thickness increases, the recovery time becomes longer, and the recovery rate becomes lower. Moreover, the PETG material performs far better than the PLA material in shape memory performance, and the greater the wall thickness, the more evident the difference between the two. When the wall thickness is 0.50 mm, the workpiece has the shortest average recovery time and the highest average recovery rate: the average recovery time for PETG is 11.70 ± 0.85 s, and the average recovery rate is 99.50 ± 0.18%; the average recovery time for PLA is 17.97 ± 1.22 s, and average recovery rate is 99 ± 0.12%. When the wall thickness is 1.50 mm, the workpiece has the longest average recovery time and lowest average recovery rate: the average recovery time for PETG is 29.28 ± 2.73 s, and the average recovery rate is 97.44 ± 0.38%; the average recovery time for PLA is 56.50 ± 4.18 s, and the average recovery rate is 96.56 ± 0.40%.

Combining with Figure 4, it can be seen that as wall thickness increases, relative density also increases, leading to excessive stress at certain nodes within the structure, resulting in an increased recovery time and decreased recovery rate of the workpiece.

### 4.3. Influence of Structural Unit Angle on Workpiece Shape Memory Performance

The structural unit angle is a key element in designing negative Poisson’s ratio honeycomb structures. It directly affects the deformation mode, rebound capability, and overall shape memory performance of the structure. Reasonable structural design can ensure that most of the energy during deformation is stored reversibly in the cellulars, thereby supporting a high shape recovery rate.

**Figure 14:**
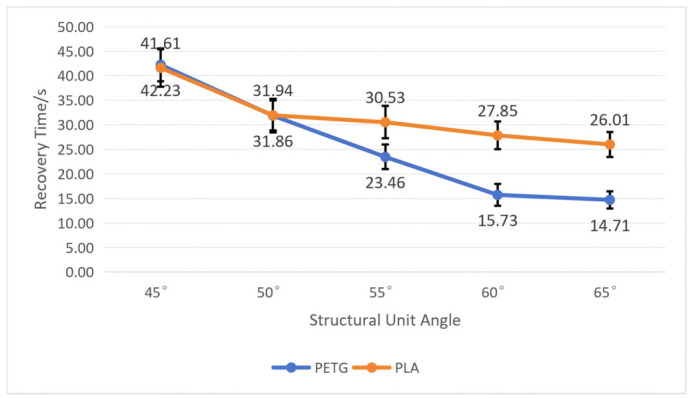
The influence of the structural unit angle on the recovery time of the workpiece (wall thickness = 1 mm; mold pressing angle = 90°). Error bars represent the standard deviation (*n* = 5).

**Figure 15:**
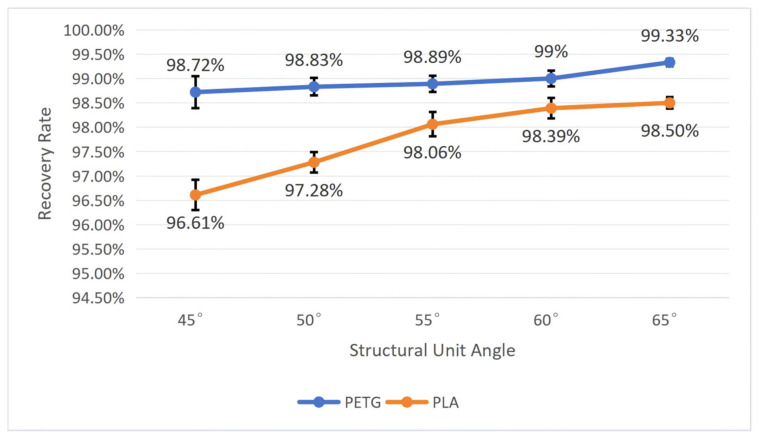
The influence of the structural unit angle on the recovery rate of the workpiece (wall thickness = 1 mm; mold pressing angle = 90°). Error bars represent the standard deviation (*n* = 5).

To test the influence of the structural unit angle on the workpiece’s shape memory performance, we fixed the wall thickness *t* at 1 mm and the mold pressing angle *θ* at 90°. By comparing workpieces with five different structural unit angles, namely, 45°, 50°, 55°, 60°, and 65°, tests were conducted on PETG and PLA workpieces, respectively, recording their recovery time and calculating their shape recovery rate according to Equation (5). After repeating each experiment five times, we calculated the average and standard deviation. The specific results are shown in Figure 14 and Figure 15.

As shown in Figure 14 and Figure 15, both materials exhibit roughly the same trend of change: as the structural unit angle increases, the recovery time becomes shorter, and the recovery rate becomes higher. Moreover, the PETG material performs far better than the PLA material in shape memory performance, and the smaller the structural unit angle, the more obvious the difference between the two. When the structural unit angle is 45°, the workpiece has the longest average recovery time and lowest average recovery rate: the average recovery time for PETG is 41.61 ± 3.36 s, and the average recovery rate is 98.72 ± 0.33%; the average recovery time for PLA is 42.23 ± 3.82 s, and the average recovery rate is 96.61 ± 0.31%. When the structural unit angle is 65°, the workpiece has the shortest average recovery time and the highest average recovery rate: the average recovery time for PETG is 14.71 ± 1.75 s, and the average recovery rate is 99.33 ± 0.8%; the average recovery time for PLA is 26.01 ± 2.55 s, and the average recovery rate is 98.50 ± 0.12%.

Combining with Figure 3, it can be seen that as the structural unit angle increases, negative Poisson’s ratio increases, leading to a greater driving force for shape recovery, thus causing a decrease in the workpiece recovery time and an increase in the recovery rate.

### 4.4. Influence of Mold Pressing Angle on Workpiece Shape Memory Performance

The mold pressing angle is the most obvious factor affecting the workpiece’s shape memory performance, and it is also an important indicator for testing the overall safety and functionality of the structure.

**Figure 16:**
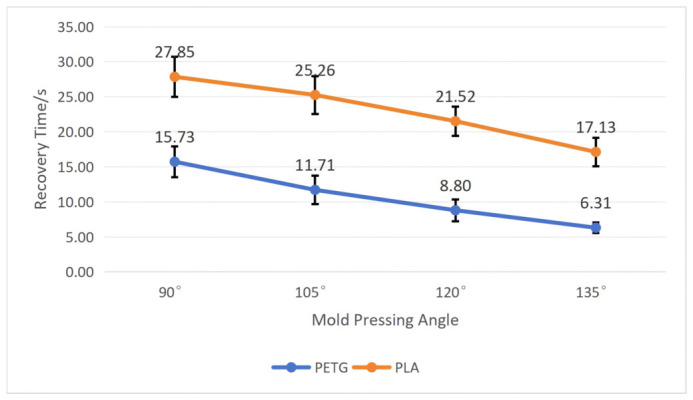
The influence of the mold pressing angle on the recovery time of the workpiece (structural unit angle = 60°; wall thickness = 1 mm). Error bars represent the standard deviation (*n* = 5).

**Figure 17:**
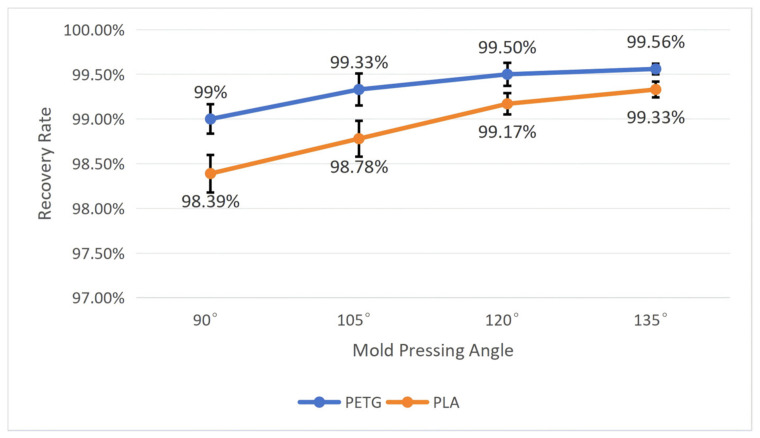
The influence of the mold pressing angle on the recovery rate of the workpiece (structural unit angle = 60°; wall thickness = 1 mm). Error bars represent the standard deviation (*n* = 5).

To test the influence of the mold pressing angle on the workpiece’s shape memory performance, we fixed the wall thickness *t* at 1 mm and the structural unit angle *α* at 60°. By comparing workpieces deformed by molds with four pressing angles, namely, 90°, 105°, 120°, and 135°, tests were conducted on PETG and PLA workpieces, respectively, recording their recovery time and calculating their shape recovery rate according to Equation (5). After repeating each experiment five times, we calculated the average and standard deviation. The specific results are shown in Figure 16 and Figure 17.

As shown in Figure 16 and Figure 17, both materials exhibit roughly the same trend of change: as the mold pressing angle increases, the recovery time becomes shorter, and the recovery rate becomes higher. Moreover, the PETG material performs far better than the PLA material in shape memory performance, and the smaller the mold pressing angle, the more obvious the difference between the two. When the mold pressing angle is 90°, the workpiece has the longest average recovery time and the lowest average recovery rate: the average recovery time for PETG is 15.73 ± 2.21 s, and the average recovery rate is 99 ± 0.17%; the average recovery time for PLA is 27.85 ± 2.85 s, and the average recovery rate is 98.39 ± 0.21%. When the mold pressing angle is 135°, the workpiece has the shortest average recovery time and the highest average recovery rate: the average recovery time for PETG is 6.31 ± 0.75 s, and the average recovery rate is 99.56 ± 0.06%; the average recovery time for PLA is 17.13 ± 2.03 s, and the average recovery rate is 99.33 ± 0.09%.

The reason for this phenomenon is that the shape recovery of 4D-printed workpieces is usually triggered by external stimuli, which need to be transmitted evenly throughout the structure. When the mold pressing angle is small, geometric distortion is minimal, internal pores and channels remain relatively unobstructed, and the paths for heat conduction or solvent diffusion are more uniform and efficient, helping the entire structure quickly and synchronously reach the critical condition for triggering phase transition, thereby achieving fast and consistent recovery. In contrast, when the mold pressing angle is large, severe geometric distortion may cause local pore closure, affecting the uniform diffusion of the stimulating medium (such as water or other solvents), leading to asynchronous recovery between cellulars, prolonging the overall recovery time, and potentially causing incomplete recovery due to local stress concentration.

### 4.5. Comparison of Shape Memory Performance Between PETG and PLA Material Workpieces

Based on the comprehensive influence of the aforementioned structural parameters on the shape memory performance of PETG and PLA workpieces, it is not difficult to see that PETG is significantly superior to PLA in both recovery time and recovery rate. This is because, at the microscopic level, as shown in the SEM images of fracture surfaces in Figure 7a,b, PETG exhibits a homogeneous amorphous structure with no distinct crystalline domains, whereas PLA displays a semi-crystalline morphology with spherulitic features. The amorphous nature of PETG provides greater chain mobility near its *T_g_* and a more pronounced entropic elastic response. Furthermore, the PETG chain contains cyclohexanedimethanol units, which increase chain flexibility. Additionally, a physical entanglement network exists between chains. The flexibility allows chain segments to easily disentangle and restore their original state upon heating, while the physical entanglement provides directionality for recovery, making the recovery process more controllable and complete. At the macroscopic level, compared to PLA, PETG possesses higher toughness, which gives PETG greater bending resistance and enables it to generate stronger recovery capability under the same stimulus. Simultaneously, PETG also has a higher *T_g_* than PLA. A higher *T_g_* means that at room temperature, the free movement of chain segments is frozen, resulting in better shape fixity. Once heated above *T_g_*, the chain segments are activated more rapidly, leading to faster recovery speed.

## 5. Response Surface Experiment

Reducing the recovery time and improving the recovery rate of workpieces have always been important research directions in 4D printing. In the previous section, the influence of wall thickness, structural unit angle, and mold pressing angle on workpiece recovery time and recovery rate was studied, and it was proven that PETG is superior to PLA in shape memory performance. To better establish the relationship between various parameters and response variables and find better parameter combinations, based on the second-order regression model of the Box–Behnken design (BBD) method within response surface methodology (RSM) [40], shape memory performance optimization was conducted on experimental samples printed under the parameter combinations designed by the Box–Behnken method [41] to obtain the relationship between design variables and objective functions [42], to optimize design variables, and to obtain the optimal parameter combination. According to the Box–Behnken experimental design principles, three factors affecting recovery time and recovery rate were selected, namely, wall thickness, structural unit angle, and mold pressing angle, each taking three levels marked as −1, 0, and 1. PETG material was used for workpiece printing. The response surface software Design Expert V25.05 was used for factor-level design, thereby obtaining the appropriate conditions for optimal recovery time and recovery rate. The factor-level table is given in Table 2.

Through the Design Expert data processing software, we input the data of each factor into the Box–Behnken data analysis software V25.05 to obtain the response surface experimental design table. Experiments were conducted according to the factor levels in the design table to obtain the experimental data results, as shown in Table 3.

### 5.1. Recovery Time and Recovery Rate Model Establishment and Analysis

The data in Table 3 were subjected to multiple regression fitting using software. Let wall thickness, structural unit angle, and mold pressing angle be *A*, *B*, and *C*, respectively. Multiple regression fitting was performed with recovery time and recovery rate as response values. The regression model coefficients and significance test results are shown in Table 4. The obtained quadratic polynomial regression models are as follows:

YRecovery Time = 20.57 + 7.53 × A − 9.76 × B − 2.46 × C + 1.67 × AB − 1.09 × AC + 4.28 × BC + 2.47 × A2 + 4.5 × B2 + 1.46 × C2

YRecovery Rate = 98.78 − 0.5 × A + 0.33 × B + 0.46 × C − 0.068 × AB + 0.21 × AC − 0.12 × BC + 0.13 × A2 + 0.21 × B2 + 0.028 × C

The *F*-value can be used to test the significance level of each variable’s influence on the response value. The larger the *F*-value, the higher the significance level of the corresponding variable. When the model’s significance test probability *p* < 0.05, the model is considered statistically significant. From Table 4, it can be seen that wall thickness, structural unit angle, and mold pressing angle all have extremely significant effects on recovery time (*p* < 0.01). The order of their influence on recovery time is *B* > *A* > *C*, i.e., structural unit angle > wall thickness > mold pressing angle. The quadratic interaction term *BC* has an extremely significant effect on recovery time (*p* < 0.01), AB has a significant effect on recovery time (*p* < 0.05), and *AC* has no significant effect on recovery time (*p* > 0.05). Therefore, the order of influence of the quadratic interaction terms on the recovery time is *BC* > *AB* > *AC*.

**Figure 18:**
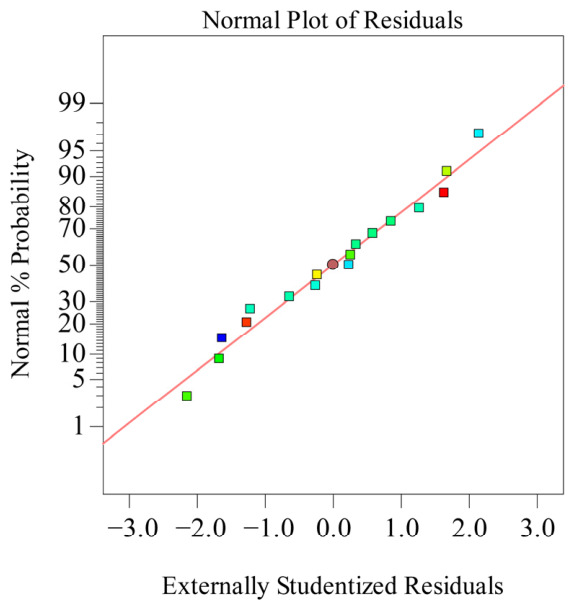
Normal distribution diagram of residuals.

**Figure 19:**
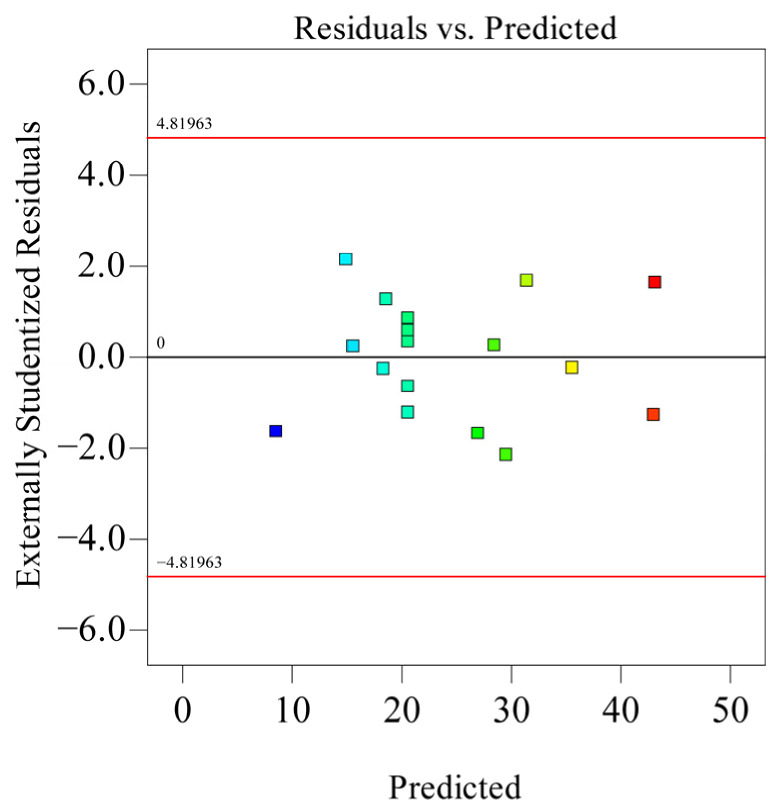
Relationship diagram between residuals and equation predictions.

**Figure 20:**
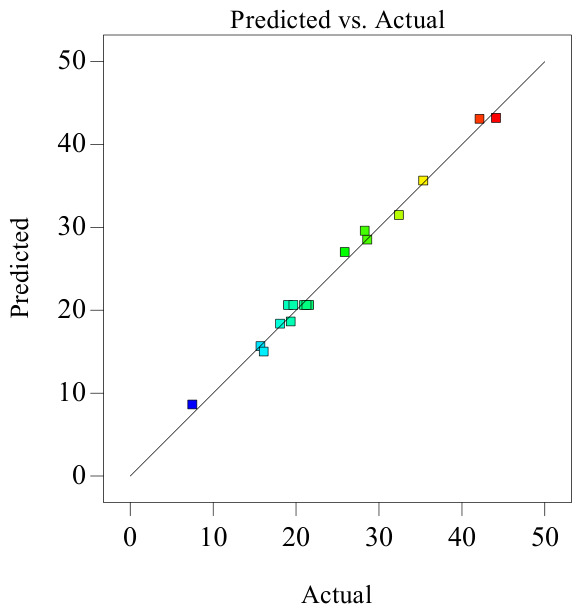
Relationship between actual and predicted values (recovery time).

As shown in Table 5, the model’s coefficient of determination *R*_2_ is 0.9910, indicating high significance of the model. Simultaneously, *Adj R*_2_ = 0.9795, explaining 97.95% of the response value variation in the experiment, and the predicted correlation coefficient *Pre R*_2_ = 0.9022 is also close. The residual normal distribution probability is shown on Figure 18. The residual normal distribution probability plot is essentially a straight line, indicating good normality of residuals and good fitting degree between the experimental model and real data. The relationship between residuals and equation-predicted values is shown in Figure 19. In Figure 19, the residuals of the 17 sets of experimental data are within ±3, indicating good data fitting. The correspondence between predicted and actual experimental values for recovery time is shown in Figure 20. As can be seen from Figure 20, the actual experimental values are distributed near the straight line, indicating little difference between actual and predicted values. This shows that the regression equation can well describe the relationship between various factors and the recovery rate. Therefore, this model can be used to analyze and predict recovery rate, and the equation has high reliability.

By analyzing the relevant data, it can be seen that the primary terms wall thickness, structural unit angle, and mold pressing angle all have extremely significant effects on recovery rate (*p* < 0.01). Analyzing the main effect relationship of each factor: *A* > *C* > *B*, i.e., wall thickness > mold pressing angle > structural unit angle. The quadratic interaction term *AC* has an extremely significant effect on recovery rate (*p* < 0.01), *BC* has a significant effect on recovery rate (*p* < 0.05), and *AB* has a significant effect on recovery rate (*p* < 0.05). Therefore, the order of influence of the quadratic interaction terms on the recovery time is *AC* > *BC* > *AB*.

**Figure 21:**
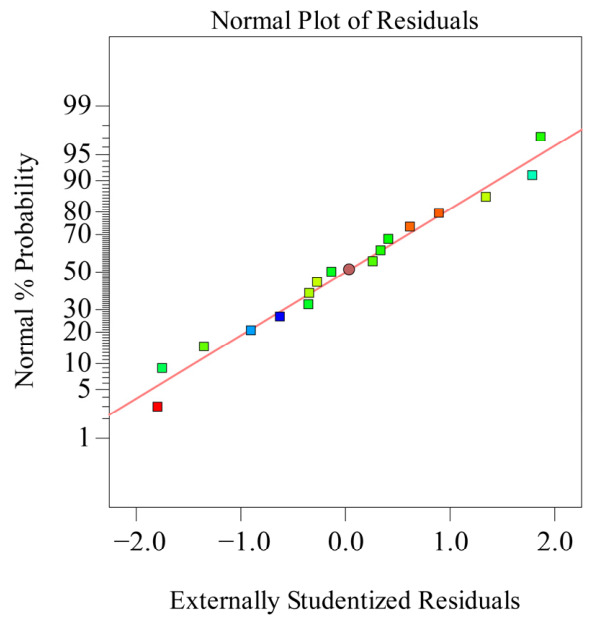
Normal distribution of residuals.

**Figure 22:**
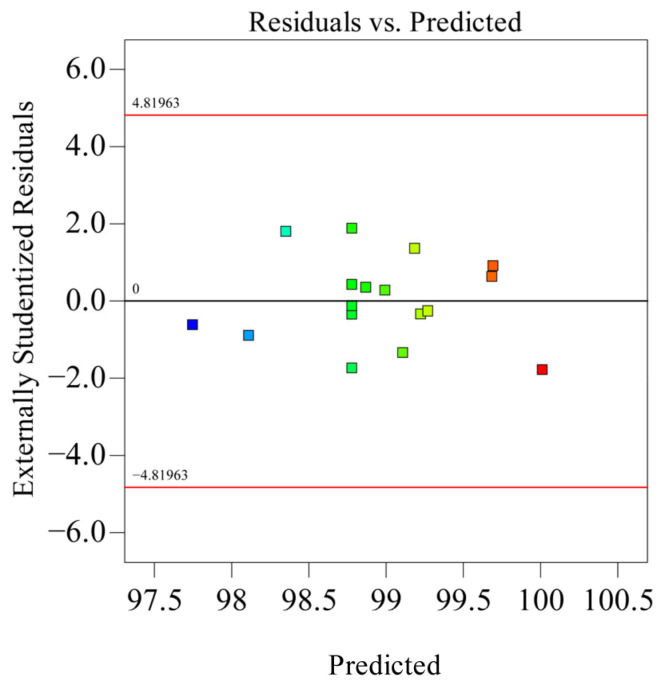
Relationship between residuals and equation predictions.

**Figure 23:**
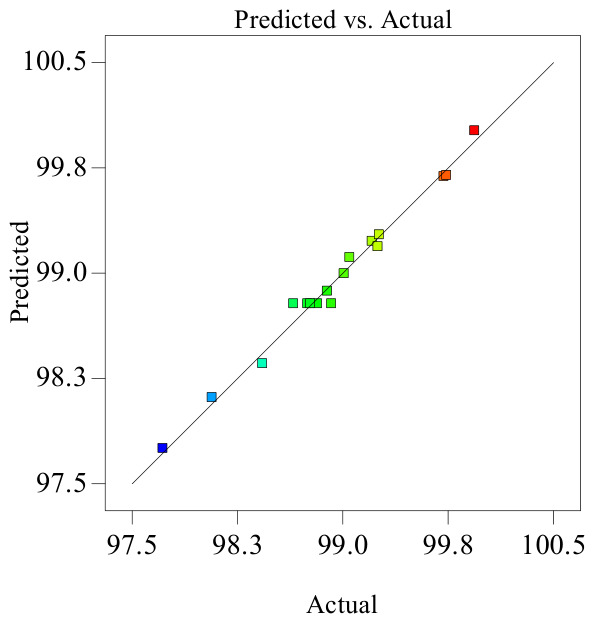
Relationship between actual and predicted values (recovery rate).

Further regression analysis was performed on this model and its regression coefficients. The results are shown in Table 6. From Table 6, it can be seen that the regression model *p* value is <0.0001 (extremely significant) and its lack of fit *p* = 0.5263 > 0.05 (not significant), indicating a good model fit, allowing for the prediction of response values using the regression equation. Simultaneously, as shown in Table 7, the model regression coefficient *R*_2_ = 0.9876, and the adjusted *R*_2_ = 0.9716, indicating that 97.16% of the data can be explained by this model, showing high equation reliability. The residual normal distribution probability plot is shown in Figure 21, the relationship between residuals and equation-predicted values is shown in Figure 22, and the correspondence between predicted and actual recovery rate values is shown in Figure 23. All three figures meet the requirements, indicating that the regression equation can well describe the relationship between various factors and the recovery rate. Therefore, this model can be used to analyze and predict recovery rate, and the equation has high reliability.

### 5.2. Interaction Between Factors

The strength of interaction between two factors is intuitively reflected by the steepness of the response surface slope and the elliptical shape of the contour lines. The steepness of the response surface and the density of the contour lines are proportional to the degree of influence of that factor on the response value. By observing the steepness of the response surface plot, the degree of influence of both on the response value can be determined.

#### 5.2.1. Influence on Workpiece Recovery Time

**Figure 24:**
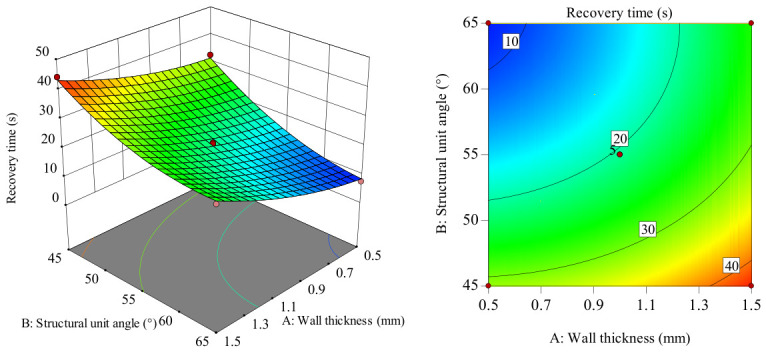
Response surface and contour plot of *AB* to recovery time.

In the intuitive diagram of the interaction between the response surface and the contour lines shown in Figure 24, when the mold pressing angle is at the zero level, the influence trend of the wall thickness–structural unit angle interaction on recovery time shows a concave surface distribution. Recovery time shows a slow increasing trend as wall thickness increases from 0.5 to 1.5 mm. Recovery time shows a decreasing trend as the structural unit angle increases from 45 to 65. Moreover, when the wall thickness values differ, the magnitude of change in recovery time with the increasing structural unit angle varies, indicating a significant interaction between wall thickness and structural unit angle. Comparatively, in the structural unit angle direction, the longitudinal span of the response surface and the gradient change in contour lines are larger, indicating that the structural unit angle has a greater influence on recovery time than wall thickness. When the wall thickness is in the range of 0.5–0.9 mm and the structural unit angle is in the range of 60–65°, recovery time can be significantly reduced.

**Figure 25:**
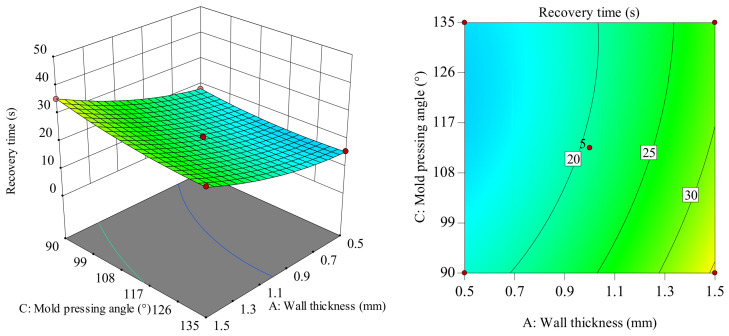
Response surface and contour plot of *AC* to recovery time.

In the intuitive diagram of the interaction between the response surface and the contour lines shown in Figure 25, when the structural unit angle is at the zero level, as the mold pressing angle increases from 90 to 135°, the recovery time results show a slowly decreasing trend. As wall thickness increases from 0.5 to 1.5 mm, recovery time shows a slowly increasing trend, and the increase in amplitude is relatively large. Considering only the influence of their interaction, the optimal process conditions for recovery time are concentrated in the combination range where wall thickness is 0.5–0.7 mm and the mold pressing angle is 117–135°. Comparatively, in the wall thickness direction, the longitudinal span of the response surface and the gradient change in the contour lines are larger, indicating that wall thickness has a greater influence on recovery time than the mold pressing angle.

**Figure 26:**
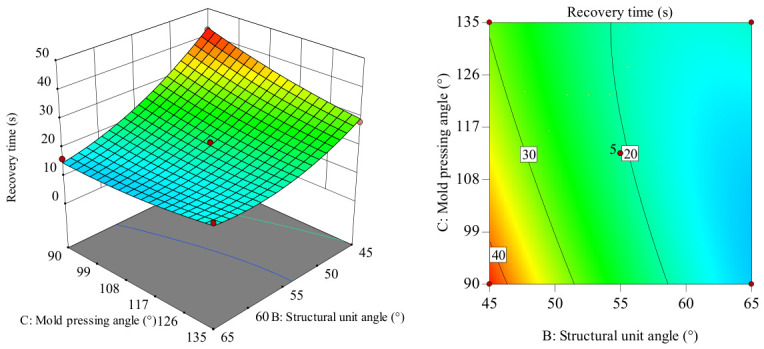
Response surface and contour map of *BC* to recovery time.

In the intuitive diagram of interaction between the response surface and the contour lines shown in Figure 26, when the wall thickness is at the zero level, recovery time shows a decreasing trend; as the structural unit angle increases from 45 to 65, the structural unit angle value is small; recovery time shows a decreasing trend as the mold pressing angle increases from 90 to 135. When the structural unit angle value is large, the recovery time shows a gentle trend as the mold pressing angle increases from 90 to 135. Considering only their interaction, when the structural unit angle is around 60–65° and the mold pressing angle is 90–108°, it is the critical optimal process parameter for recovery time. Comparatively, in the structural unit angle direction, the longitudinal span of the response surface and the gradient change in the contour lines are larger, indicating that the structural unit angle has a greater influence on recovery time than the mold pressing angle.

In summary, regarding the influence on workpiece recovery time: structural unit angle > wall thickness > mold pressing angle.

#### 5.2.2. Influence on Workpiece Recovery Rate

**Figure 27:**
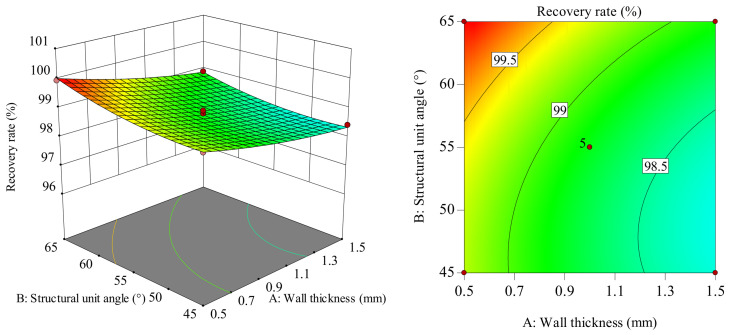
Response surface and contour plot of *AB* to recovery rate.

The influence of the interaction between the wall thickness and the structural unit angle on the recovery rate is shown in Figure 27. In the AB interaction surface, as wall thickness increases from 0.5 to 1.5 mm, recovery rate gradually decreases. The slope of the recovery rate shows a slowly increasing trend as the structural unit angle increases from 45 to 65°. Considering only their interaction, when the wall thickness is around 0.5–0.7 mm and the structural unit angle is in the range of 60–65°, the recovery rate reaches its maximum value. Comparatively, the recovery rate fluctuates more in the wall thickness direction, indicating that the wall thickness has a greater influence on the recovery rate than the structural unit angle. The surface plot is also consistent with the variance analysis results in Table 6.

**Figure 28:**
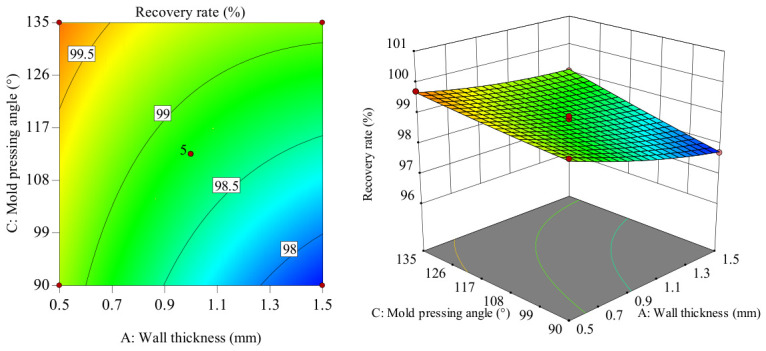
Response surface and contour plot of *AC* to recovery rate.

The influence of the interaction between the wall thickness and the mold pressing angle on the recovery rate is shown in Figure 28. In the *AC* interaction surface, the slope of the recovery rate change shows a decreasing trend as the wall thickness increases from 0.5 to 1.5 mm. When the wall thickness value is small, the slope of the recovery rate change shows a gradually increasing trend as the mold pressing angle increases from 90 to 135°, with a small increase in amplitude. When the wall thickness value is large, the slope of the recovery rate change shows a gradually increasing trend as the mold pressing angle increases from 90 to 135°, with a large increase in amplitude. This indicates a significant interaction between wall thickness and mold pressing angle. Considering only their interaction, when wall thickness is 0.5–0.7 mm and mold pressing angle is in the range of 117–135°, the recovery rate reaches its maximum value. Comparatively, the recovery rate fluctuates more in the wall thickness direction, indicating that the wall thickness has a greater influence on the recovery rate than the mold pressing angle. The surface plot is also consistent with the variance analysis results in Table 6.

**Figure 29:**
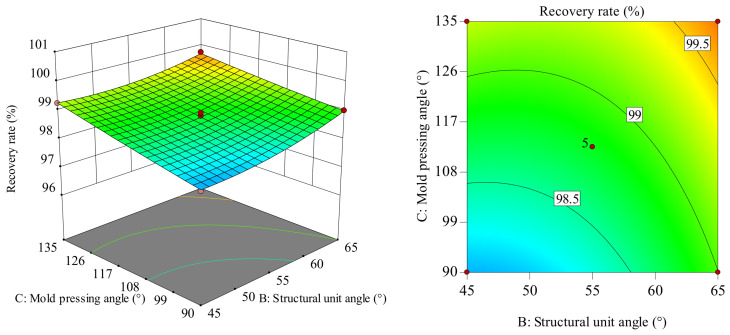
Response surface and contour plot of *BC* to recovery rate.

The influence of the interaction between the structural unit angle and the mold pressing angle on the recovery rate is shown in Figure 29. In the *BC* interaction surface, the slope of the recovery rate change shows an increasing trend as the mold pressing angle increases from 90 to 135. The slope of the recovery rate change shows a slowly increasing trend as the structural unit angle increases from 45 to 65. Moreover, when the mold pressing angle values differ, the amplitude of change in the recovery rate slope with an increasing structural unit angle varies, indicating a significant interaction between the structural unit angle and the mold pressing angle. Considering only their interaction, when the structural unit angle is 60–65° and the mold pressing angle is in the range of 117–135°, the recovery rate reaches its maximum value. Comparatively, the recovery rate fluctuates more in the mold pressing angle direction, indicating that the mold pressing angle has a greater influence on the recovery rate than the structural unit angle. The surface plot is also consistent with the variance analysis results in Table 6.

In summary, regarding the influence on workpiece recovery rate: wall thickness > mold pressing angle > structural unit angle.

### 5.3. Experimental Result Verification

According to the regression equation model, with the minimum recovery time and maximum recovery rate as the optimization objectives, the predicted optimal conditions are a wall thickness of 0.5 mm, a structural unit angle of 65°, and a mold pressing angle of 104.44°. Due to limitations in mold fabrication, it was not possible to prepare a mold with the exact predicted angle of 104.44°. Therefore, validation experiments were conducted under the nearest achievable angle of 105° (using an adjustable mold) and, for comparison, under the maximum angle of 135° tested in the response surface design, as this angle also fell within the validated parameter range. Three parallel tests were performed for each condition. Under the 105° condition, the measured recovery time was 7.586 ± 0.163 s, and the recovery rate was 99.93 ± 0.037%. Under the 135° condition, the recovery time was 7.486 ± 0.172 s, and the recovery rate was 99.94 ± 0.026%. Compared with the predicted values for the total recovery time (7.721 s) and the recovery rate (99.97%), both sets of experimental results showed that the difference is within 5%, confirming the reliability of the response surface model.

As shown in Table 8, considering the discrete availability of the mold angles and the slightly better mean performance observed at 135°, this condition was selected as the final validation point. The high consistency between the predicted and experimental outcomes demonstrates that the optimized parameters obtained by response surface methodology are reasonable and effective.

## 6. Conclusions

A re-entrant negative Poisson’s ratio honeycomb structure with periodic arrangement characteristics was designed. The samples were successfully fabricated via 3D modeling and 3D printing, and shape memory performance tests were conducted. The following conclusions are drawn.

The relative density of the negative Poisson’s ratio honeycomb structure is mainly influenced by the cellular wall thickness, increasing with increasing wall thickness. Its negative Poisson’s ratio is mainly influenced by the angle between the horizontal and inclined cellular walls, i.e., the structural unit angle, increasing with increasing structural unit angle.

(1) The shape memory performance of the negative Poisson’s ratio honeycomb structure is mainly related to the wall thickness and structural unit angle among the cellular parameters. The larger the wall thickness, the worse the shape memory performance; the larger the structural unit angle, the better the shape memory performance.
(2) Under the specific re-entrant hexagonal negative Poisson’s ratio configuration and thermal activation conditions employed in this study, PETG demonstrated superior shape memory performance compared to PLA, achieving both shorter recovery times and higher recovery rates. This suggests that PETG holds significant promise for 4D printing applications requiring rapid and complete shape recovery, particularly in structures with negative Poisson’s ratio characteristics.
(3) Through response surface experimental design, it was found that regarding the influence on workpiece recovery time, structural unit angle > wall thickness > mold pressing angle; regarding the influence on workpiece recovery rate, wall thickness > mold pressing angle > structural unit angle.
(4) Through response surface methodology optimization and actual experimental verification, the optimal parameters of the negative Poisson’s ratio honeycomb structure to achieve a minimum recovery time and maximum recovery rate are wall thickness of 0.5 mm, a structural unit angle of 65°, and a mold pressing angle of 135°. The error compared to the predicted values does not exceed 5%.

## Figures and Tables

**Table 1:** Test piece printing process parameters.

Type of Material	PETG	PLA
Nozzle Temperature/°C	245 °C	220 °C
Texture PEI Print Board Temperature/°C	70 °C	55 °C
Number of Storeys of Single-Layer Wall	2	2
Printing Layer Velocity/(mm/s)	50	50
Floor Height/mm	0.2	0.2
Line Width/mm	0.42	0.42
Nozzle Diameter/mm	0.4	0.4
Density of Filling	15%	15%
Fill Pattern	Gird Shape	Gird Shape
Printing Direction	Horizontal	Horizontal

**Table 2:** Surface analysis experimental design table with three factors and three horizontal responses.

Factor	Horizontal		
	−1	0	1
A—Wall Thickness (mm)	0.5	1.0	1.5
B—Structural Unit Angle (°)	45	55	65
C—Mold Pressing Angle (°)	90	112.5	135

**Table 3:** Experimental design and results of response surface optimization recovery time and recovery rate.

Number	A—Wall Thickness (mm)	B—Structural Unit Angle (°)	C—Mold Pressing Angle (°)	Y_1_—Recovery Time (s)	Y_2_—Recovery Rate (%)
1	0.5	45	112.5	32.47	99.21
2	1.5	45	112.5	44.18	98.43
3	0.5	65	112.5	7.56	99.94
4	1.5	65	112.5	25.94	98.89
5	0.5	55	90	18.14	99.25
6	1.5	55	90	35.41	97.72
7	0.5	55	135	15.77	99.72
8	1.5	55	135	28.66	99.05
9	1	45	90	42.19	98.07
10	1	65	90	16.16	99.01
11	1	45	135	28.34	99.26
12	1	65	135	19.43	99.74
13	1	55	112.5	19.11	98.75
14	1	55	112.5	21.02	98.82
15	1	55	112.5	21.65	98.65
16	1	55	112.5	19.74	98.92
17	1	55	112.5	21.33	98.77

**Table 4:** Regression analysis results of recovery time model and regression coefficients.

Source	Sum of Squares	*df*	Mean Square	*F*-Value	*p*-Value	Level of Significance
Model	1484.31	9	164.92	85.77	< 0.0001	**
*A*—Wall Thickness	453.76	1	453.76	235.99	< 0.0001	**
*B*—Structural Unit Angle	762.26	1	762.26	396.43	< 0.0001	**
*C*—Mold Pressing Angle	48.51	1	48.51	25.23	0.0015	**
*AB*	11.12	1	11.12	5.78	0.0471	*
*AC*	4.80	1	4.80	2.49	0.1583	
*BC*	73.27	1	73.27	38.11	0.0005	**
*A* ^2^	25.61	1	25.61	13.32	0.0082	**
*B* ^2^	85.31	1	85.31	44.37	0.0003	**
*C* ^2^	8.96	1	8.96	4.66	0.0677	**
Residual	13.46	7	1.92			
Lack of Fit	8.69	3	2.90	2.43	0.2053	ns
Pure Error	4.77	4	1.19			
Cor Total	1497.77	16				

Note: *p* < 0.01 is extremely significant, expressed as **. *p* < 0.05 is significant, expressed as *. *p* > 0.05 is not significant, expressed as ns.

**Table 5:** Results of error statistical analysis of recovery time regression model.

Project	Value	Project	Value
Standard Deviation	1.39	Model Correlation Coefficient (*R*^2^)	0.9910
Mean Value	24.54	Correction Decision Coefficient (*R*^2^*_Adj_*)	0.9795
Coefficient of Variation/%	5.65	Prediction Decision Coefficient (*R*^2^*_Pre_*)	0.9022
Sum of Squares of Predicted Errors	146.53	Relative Accuracy Degree	32.520

**Table 6:** Regression analysis results of recovery rate model and regression coefficients.

Source	Sum of Squares	*df*	Mean Square	*F*-Value	*p*-Value	Level of Significance
Model	5.14	9	0.57	61.89	<0.0001	**
*A*—Wall Thickness	2.03	1	2.03	219.96	<0.0001	**
*B*—Structural Unit Angle	0.85	1	0.85	92.26	<0.0001	**
*C*—Mold Pressing Angle	1.73	1	1.73	187.43	<0.0001	**
*AB*	0.018	1	0.018	1.97	0.2027	
*AC*	0.18	1	0.18	20.03	0.0029	**
*BC*	0.053	1	0.053	5.73	0.0479	*
*A* ^2^	0.066	1	0.066	7.16	0.0318	*
*B* ^2^	0.19	1	0.19	20.17	0.0028	**
*C* ^2^	0.003242	1	0.003242	0.35	0.572	
Residual	0.065	7	0.009229			
Lack of Fit	0.026	3	0.008508	0.87	0.5263	ns
Pure Error	0.039	4	0.00977			
Cor Total	5.21	16				

Note: *p* < 0.01 is extremely significant, expressed as **. *p* < 0.05 is significant, expressed as *. *p* > 0.05 is not significant, expressed as ns.

**Table 7:** Results of error statistical analysis of recovery rate regression model.

Project	Value	Project	Value
Model Correlation Coefficient (*R*^2^)	0.096	Model Correlation Coefficient (*R*^2^)	0.9876
Correction Decision Coefficient (*R*^2^*_Adj_*)	98.95	Correction Decision Coefficient (*R*^2^*_Adj_*)	0.9716
Prediction Decision Coefficient (*R*^2^*_Pre_*)	0.097	Prediction Decision Coefficient (*R*^2^*_Pre_*)	0.9098
Relative Accuracy Degree	0.47	Relative Accuracy Degree	30.723

**Table 8:** Validation results of response surface optimization.

Parameter	Predicted Value	Validation Condition	Experimental Value (Mean ± SD, *n* = 3)	Relative Error (%)
Recovery Time (s)	7.72	105°	7.586 ± 0.163	1.8
		135°	7.486 ± 0.172	3.1
Recovery Rate (%)	99.97	105°	99.93 ± 0.037	0.04
		135°	99.94 ± 0.026	0.03

## Data Availability

The raw data supporting the conclusions of this article will be made available by the authors on request.
